# Preparation and Thermal Performance Enhancement of Low Temperature Eutectic Composite Phase Change Materials Based on Na_2_SO_4_·10H_2_O

**DOI:** 10.3390/ma11112230

**Published:** 2018-11-09

**Authors:** Pumin Hou, Jinfeng Mao, Fei Chen, Yong Li, Xian Dong

**Affiliations:** College of Defense Engineering, Army Engineering University of PLA, Nanjing 210007, China; lgdxhpm@163.com (P.H.); xmbqchen@outlook.com (F.C.); jessicadx1221@163.com (X.D.)

**Keywords:** Na_2_SO_4_·10H_2_O–KCl eutectics, composite phase change materials, thermal performance, thermal reliability

## Abstract

In this paper, a series of Na_2_SO_4_·10H_2_O–KCl eutectic mixtures were prepared by adding different mass fractions of KCl (1 wt.%, 3 wt.%, 5 wt.%, or 7 wt.%) to Na_2_SO_4_·10H_2_O. Polyacrylamide (PAM) was proposed as the thickener, sodium tetraborate decahydrate (STD) was proposed as the nucleating agent, and expanded graphite (EG) was proposed as the high thermal conductivity medium for Na_2_SO_4_·10H_2_O–5 wt.% KCl eutectics. The results showed that in Na_2_SO_4_·10H_2_O–5 wt.% KCl eutectics with 5 wt.% PAM and 5 wt.% STD, almost no phase separation occurred, and the degree of supercooling was reduced to 0.4 °C. The thermal performance of Na_2_SO_4_·10H_2_O–5 wt.% KCl composite phase change materials (CPCMs) with varying contents of EG was explored. The results showed that EG could improve the thermal conductivity effectively and that the mass fraction of EG should be no more than 3%, otherwise the crystallization value and supercooling would deteriorate. The thermal reliability of the Na_2_SO_4_·10H_2_O–5 wt.% KCl eutectic CPCMs containing 5 wt.% PAM, 5 wt.% STD, and 3 wt.% EG was investigated, mainly through the ambient temperature, thermal cycling test, and TGA analysis. The results demonstrated that these CPCMs showed perfect thermal reliability.

## 1. Introduction

Building energy consumption accounts for an increasing proportion of global energy consumption year by year (up to 40%), while heating, ventilation, and air conditioning (HVAC) systems account for 60% of building energy consumption [1]. Therefore, reducing the energy consumption of HVAC systems is one of the most important ways to cut down building energy consumption and global energy consumption. Latent energy storage technology (LEST), which can improve the thermal inertia of building envelopes, reduce indoor temperature fluctuations, improve personnel thermal comfort, and are becoming an effective way to reduce reliance on traditional HVAC systems [2,3]. As the core of the LEST, the phase change materials (PCMs) used in building energy efficiency have been widely studied [4,5]. Compared with traditional building materials, PCMs can store energy through the utilization of sensible and latent heat and require only about 6% of the thickness of a traditional cement wall, which saves building materials [6].

The PCMs selected for building envelopes should possess a suitable melting point, high heat storage density, good thermal conductivity, small volume change, and so on [1,7]. These materials mainly include paraffin waxes [8,9,10], fatty acids [11], salt hydrates [12,13], and eutectics [14,15]. The single-component organic or inorganic PCM has a fixed melting point, which makes it difficult to meet the requirements of building in different climate zones. Eutectics can be achieved by mixing two or more PCMs. By controlling the ratio between the different components, a series of PCMs with different melting points can be prepared, which provides more options for their application.

The melting point of the eutectics used in the building envelope is mostly between 15 and 30 °C [1], which provides a comfortable thermal environment for occupants. The indoor air temperature when using a PCM can be reduced by up to 4.2 °C [16]. The maximum time delay of the peak heat flux/temperature is about 6 h [17]. The melting temperature of the selected PCMs should be matched to the desired operating temperature. Researchers have conducted extensive research on eutectic PCMs suitable for building maintenance structures, mainly including paraffin eutectics [18,19], fatty acid eutectics [20,21,22], and hydrated salt eutectics [23] among others. Hydrated salt eutectics, which have a high latent heat and good heat transfer performance, are suitable for low-temperature heat storage and have been widely studied [24,25]. Na_2_SO_4_·10H_2_O has received extensive attention as a potential hydrated salt PCM [26]. However, there have only been a few reports on the preparation of eutectic mixtures based on Na_2_SO_4_·10H_2_O.

In this paper, a series of low-temperature eutectic composite phase change materials (CPCMs) consisting of Na_2_SO_4_·10H_2_O and KCl were prepared. The Na_2_SO_4_·10H_2_O–KCl eutectics were modified by adding polyacrylamide (PAM; a thickener), sodium tetraborate decahydrate (STD; a nucleating agent), and expanded graphite (EG; a high thermal conductivity medium). Then, the phase separation condition, cooling curve, differential scanning calorimeter (DSC) curve, and thermal conductivity of the Na_2_SO_4_·10H_2_O eutectic CPCMs were explored, and the corresponding thermophysical parameters were obtained. The thermal performance of the Na_2_SO_4_·10H_2_O eutectic CPCMs were tested, including the external ambient temperature test, thermal cycling stability test, and TGA test.

## 2. Materials and Methods

### 2.1. Materials

Sodium sulfate decahydrate (Na_2_SO_4_·10H_2_O, analytical reagent (AR), purity >99%), potassium chloride (KCl, AR, purity >99%), sodium tetraborate decahydrate (Na_2_B_4_O_7_·10H_2_O, AR, purity >99%), and polyacrylamide ((C_3_H_5_NO)_n_, solid content >90%, molecular weight ≥10 million) were offered by the Sinopharm Chemical Reagent Co., Ltd. (Shanghai, China). Expanded graphite (expanded ratio: 234 mL/g, carbon content: 99%) was purchased from the Qingdao Taixing Graphite Co., Ltd. (Qingdao, China).

### 2.2. Preparation of Na_2_SO_4_·10H_2_O Composites

The eutectic CPCMs were obtained by a simple blending method. The Na_2_SO_4_·10H_2_O was melted in a thermostatic water bath at 40 °C, followed by the addition of KCl (1 wt.%, 3 wt.%, 5 wt.%, or 7 wt.%). PAM was selected as the thickener, and STD was selected as the nucleating agent for the Na_2_SO_4_·10H_2_O–KCl. All sample numbers and configurations are shown in Table 1. Lastly, EG (1–5 wt.%) was added to the above optimum proportioning composites with no subcooling and phase separation. In order to make sure that the CPCMs were uniformly mixed, the samples were stirred homogeneously at 40 °C for 20 min and ultrasonically dispersed for 30 min.

### 2.3. Characterization

#### 2.3.1. Cooling Curve Test

The sample was placed and melted in a 5 mL centrifuge tube, and a T-type thermocouple was then inserted in the melted PCMs. The thermocouple was located in the middle of the tube, 10 mm from the bottom, and was fixed during the test. The tube was heated from an initial temperature (5 or 15 °C) by a metal bath. After the PCMs reached the set heating temperature of the metal bath, heating was continued for at least 10 min to ensure that the PCMs were fully melted. Then, the tube was placed and cooled in another metal bath at the initial temperature. The temperature inside the tube during the test was recorded automatically by a data acquisition system, with an interval of 9 s. The cooling curves were obtained by collating the monitored temperature data.

#### 2.3.2. DSC Analysis

The thermal properties, such as the phase transition temperature and enthalpy, were tested by a DSC tester (TA Q200, New Castle, DE, USA) under a constant nitrogen flow of 50 mL/min at a heating rate of 20 °C/min, and the temperature varied from −10 °C to 50 °C. Each test sample weighed about 10 mg and was tableted using the tablet press (T-Zero).

#### 2.3.3. Thermal Conductivity Test

The thermal conductivity of the samples was determined by the transient plane source method with a thermal conductor tester (DZDR-S, Nanjing, China) for 40 s at 0.5 W, at room temperature. The samples were prepared as cubes with dimensions of 50 × 50 × 30 mm^3^.

#### 2.3.4. TGA Analysis

The thermal stability of the Na_2_SO_4_·10H_2_O eutectic CPCMs was measured by the thermogravimetric analyzer (TA Q500, New Castle, DE, USA) under a constant argon flow of 40 mL/min. The heating rate was 10 °C/min, and the temperature varied from 30 °C to 120 °C. Each test sample weighed about 10 mg.

## 3. Results and Discussion

### 3.1. Properties of the Na_2_SO_4_·10H_2_O–KCl Eutectic Mixture

**Figure 1:**
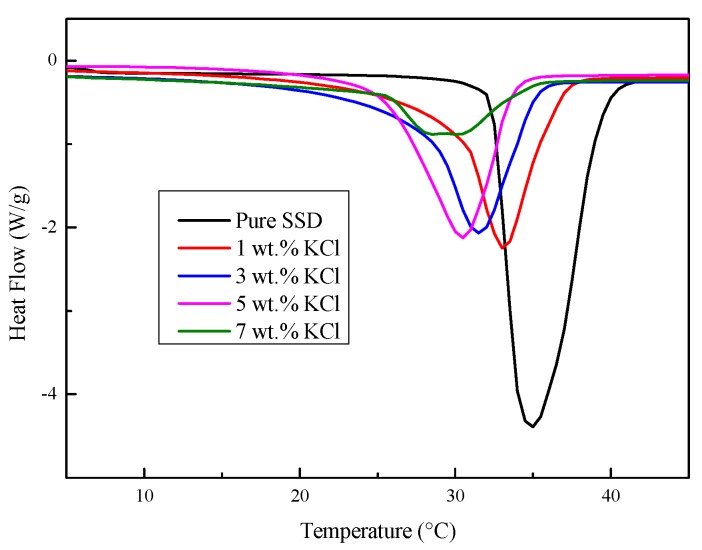
DSC curves of the Na_2_SO_4_·10H_2_O–KCl eutectic mixtures with varying contents of KCl.

DSC curves of the Na_2_SO_4_·10H_2_O–KCl eutectic mixtures with varying contents of KCl (0 wt.%, 1 wt.%, 3 wt.%, 5 wt.%, and 7 wt.%) are presented in Figure 1, and the corresponding thermal properties are shown in Table 2. It can be seen from Figure 1 that the DSC curves and peak temperatures of the Na_2_SO_4_·10H_2_O–KCl eutectic CPCMs shifted to the left as the KCl mass fraction increased, which indicated that the mixtures could be melted at a lower temperature and store latent heat. As shown in Table 2, both the melting temperature and enthalpy of the eutectic CPCMs decreased as the KCl mass fraction increased. This was because the Na_2_SO_4_·10H_2_O was affected by the new structure formed between K^+^ ions and the original ion clusters, and the new sharing ion pairs and contact ion pairs led to a reduction in the melting temperature and enthalpy [27].

**Figure 2:**
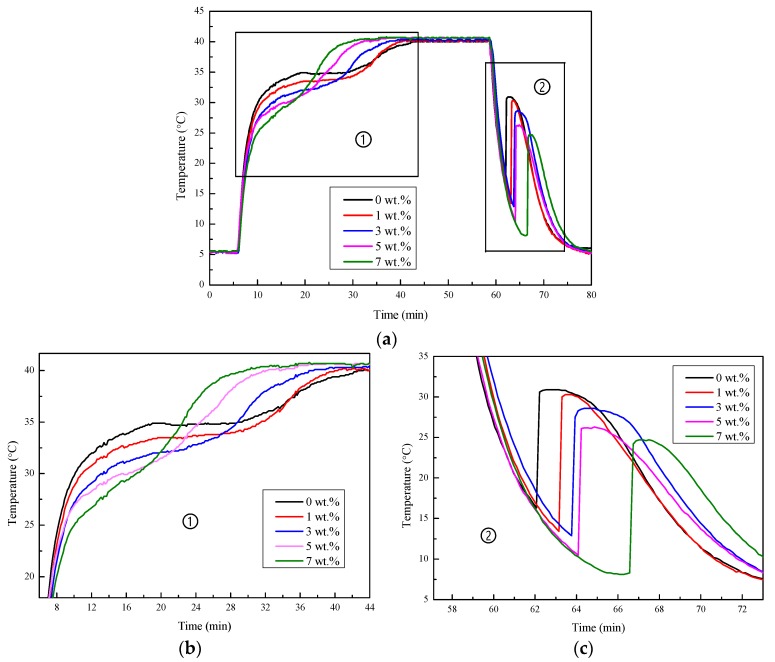
Cooling curves of the Na_2_SO_4_·10H_2_O–KCl eutectic mixtures with varying contents of KCl: (**a**) whole cooling curves; (**b**) enlargement of the heating process; (**c**) enlargement of the cooling process.

Figure 2 shows the cooling curves of the Na_2_SO_4_·10H_2_O–KCl eutectic mixtures. The test process was divided into three stages, and the heating and the cooling temperatures of the metal bath were set to 40 °C and 5 °C, respectively.

The first stage was the heating process. The temperature of the sample increased dramatically after being heated from the initial temperature. When the sample reached the melting temperature, a latent heat storage platform appeared and then rapidly rose to the set temperature. From Figure 2 and the partial enlargement of the heating process (Figure 2b), it can be seen that the slope of the temperature curve showed few differences at an earlier stage. As the temperature increased, the curves began to separate; the larger the KCl mass fraction in the sample, the earlier the curves separated. This was because the melting temperature of the Na_2_SO_4_·10H_2_O–KCl eutectic mixture decreased as the KCl content increased, which led to the earlier start of the latent heat storage process, and the curve became more gentle. After the end of the latent heat storage process, the temperature of the samples increased rapidly and exceeded the material with a lower KCl content. It can be noted that, with the increase in KCl mass fraction, the latent heat storage platform was maintained for a shorter period of time and the inclination of the platform was greater. This was because the enthalpy of the sample was reduced, and the heat storage capacity weakened as the KCl content increased. The ability of the PCM to control temperature was therefore weakened.

In the second stage, the sample was kept at 40 °C for about 20 min to ensure it was sufficiently melted to prevent unmelted crystal from acting as a crystal nucleus.

The third stage was the cooling process. The temperature of the sample decreased sharply after being cooled from roughly 40 °C. When the sample reached the minimum temperature (crystallization starting temperature), the samples began to crystallize and then rapidly rose to the crystallization temperature. After the end of the latent heat release platform, the temperature gradually reduced to the cooling temperature. The difference between the crystallization temperature and the crystallization starting temperature is taken as the degree of supercooling [28]. From the partial enlargement of the cooling process (Figure 2c), it can be seen that KCl had a significant effect on the crystallization starting temperature and crystallization temperature of the Na_2_SO_4_·10H_2_O–KCl eutectic mixtures. The detailed data are shown in Table 2. Compared with pure Na_2_SO_4_·10H_2_O, the crystallization starting temperature and crystallization temperature of the mixtures decreased as the KCl increased, especially in the composites containing 7 wt.% KCl—from 16.3 to 30.9 °C down to 8.3 and 24.7 °C. At the same time, it can be seen that the degree of supercooling of the Na_2_SO_4_·10H_2_O had not decreased. This was because KCl could not act as the nucleating agent, and the power required for crystallization did not decrease. This indicates that although KCl could effectively reduce the melting temperature of Na_2_SO_4_·10H_2_O, it could not reduce the degree of supercooling.

### 3.2. Properties of Na_2_SO_4_·10H_2_O–5 wt.% KCl Eutectics Containing PAM and STD

#### 3.2.1. Phase Separation

**Figure 3:**
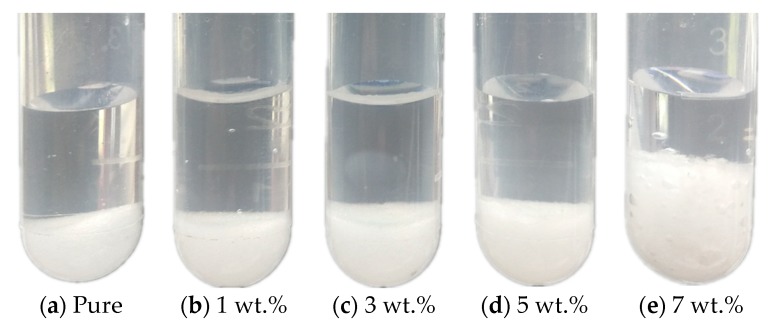
Photographs of the Na_2_SO_4_·10H_2_O–KCl eutectics containing different mass fractions of KCl: (**a**–**e**) 0–7 wt.%.

Figure 3 shows the state of the Na_2_SO_4_·10H_2_O–KCl eutectics containing different mass fractions of KCl. According to Figure 3, all of the pure Na_2_SO_4_·10H_2_O and eutectics with varying contents of KCl showed serious phase separation. This was because Na_2_SO_4_·10H_2_O was dehydrated by heating and formed Na_2_SO_4_ anhydrous salt, which exhibits low solubility and high density, and was deposited to the bottom of the test tube. Phase separation resulted in the Na_2_SO_4_ in the bottom being unable to combine with the water on the upper layer, which led to the reduction in the heat storage capacity. According to the analysis above, Na_2_SO_4_·10H_2_O–KCl eutectics suffered serious subcooling and phase separation and therefore needed to be modified.

**Figure 4:**
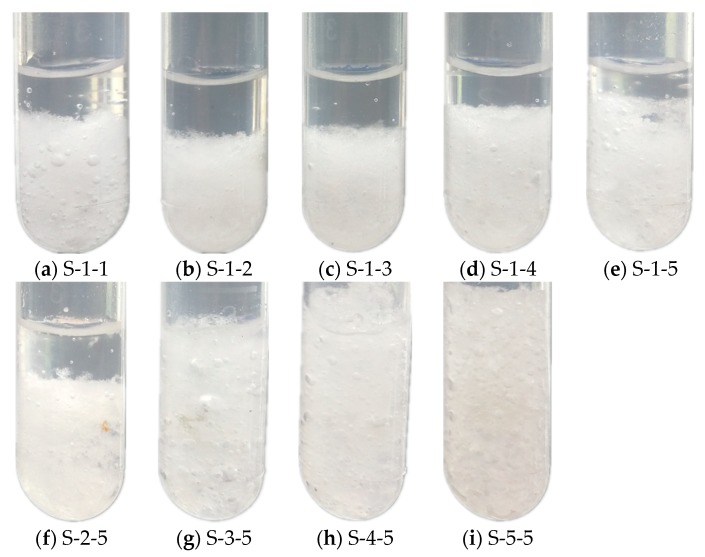
Photographs of the Na_2_SO_4_·10H_2_O–5 wt.% KCl eutectics containing varying contents of PAM and STD: (**a**–**e**) S-1-1–S-1-5; (**f**–**i**) S-2-5–S-5-5.

PAM was selected as the thickener, and STD was selected as the nucleating agent for Na_2_SO_4_·10H_2_O–KCl. The eutectic with 5 wt.% KCl was selected, and the experimental scheme is given in Table 1. The phase separation photographs are presented in Figure 4. It was determined that the material in the test tube was obviously layered when the addition of PAM was no more than 2 wt.%. It was noticed that the phase separation was relieved, and the ratio of clear liquid decreased with the increase in nucleating agent content. This was due to the addition of a nucleating agent that made the inorganic salt and liquid water easier to combine, which was beneficial to crystal growth and improved the crystallization proportion. However, because of the low content of the thickener, the viscosity of the solution was insufficient, and the phase separation phenomenon still occurred. When the content of PAM added was 3 wt.%, a small amount of liquid appeared at the top of the test tube, and the phase separation phenomenon was remarkably alleviated. As the PAM addition increased to more than 3 wt.%, the phase separation phenomenon disappeared and the materials became uniformly mixed.

#### 3.2.2. Thermal Storage and Release Time

**Figure 5:**
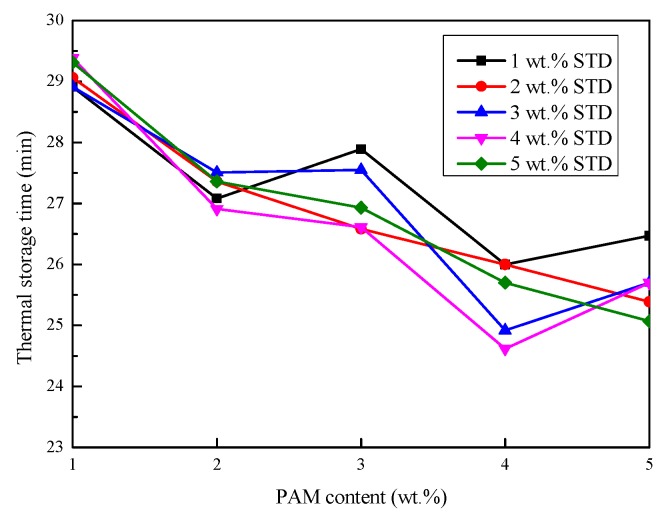
Effect of PAM and STD content on the thermal storage time of the Na_2_SO_4_·10H_2_O–5 wt.% KCl eutectics.

**Figure 6:**
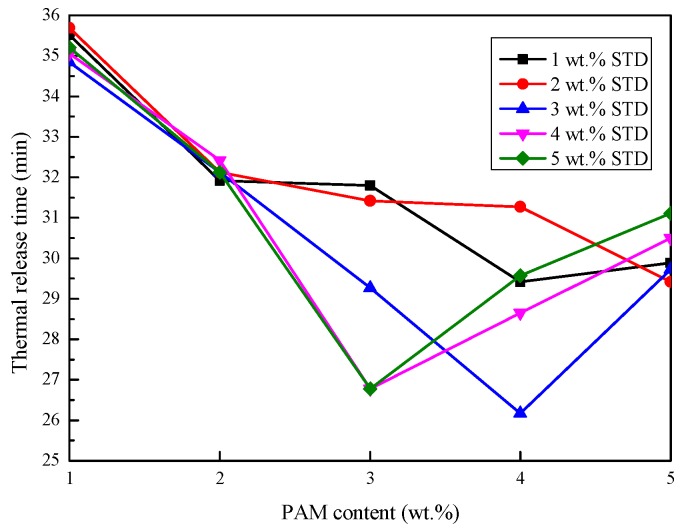
Effect of PAM and STD content on the thermal release time of the Na_2_SO_4_·10H_2_O–5 wt.% KCl eutectics.

The cooling curves were analyzed for all samples and are shown in Table 1. The time required for heating the sample from 15 °C to 40 °C was taken as the thermal storage time, and the time required for the inverse process was taken as the thermal release time. The thermal storage and release time of the Na_2_SO_4_·10H_2_O–5 wt.% KCl eutectics containing different proportions of PAM and STD are presented in Figure 5 and Figure 6. It can be seen from Figure 5 that the thermal storage time of Na_2_SO_4_·10H_2_O composites decreased with the increase in the PAM mass fraction. This was because PAM was an impurity in Na_2_SO_4_·10H_2_O and caused a slight attenuation of enthalpy.

Compared with the thermal storage stage, the thermal release stage had problems such as subcooling and phase separation. Therefore, the thermal release time curves of the composites were not exactly the same as the thermal storage time curves. The effect of STD content on the thermal release time under the same ratio of PAM was analyzed, and it was found that when the proportion of PAM was 1 wt.% and 2 wt.%, STD had little effect on the thermal release time. This was because insufficient PAM caused the phase separation of the Na_2_SO_4_·10H_2_O, and even when the STD content was large enough, the Na_2_SO_4_·10H_2_O could not crystallize well.

In analyzing the effect of PAM content on the thermal release time under the same ratio of STD, it was found that when the proportion of STD was 1 wt.% and 2 wt.%, the thermal release time decreased with the increase in the PAM mass fraction. This was because a deficiency of the nucleating agent caused the Na_2_SO_4_·10H_2_O to be unable to nucleate completely. When the proportion of STD exceeded 2 wt.%, the thermal release time curve decreased initially, followed by an increase when the PAM mass fraction increased. This was because the Na_2_SO_4_·10H_2_O was partially crystallized when the PAM was insufficient. As the PAM mass fraction was increased to a certain proportion, the phase separation disappeared and the Na_2_SO_4_·10H_2_O fully crystallized, which extended the thermal release process. The inflection point at which the thermal release process began to increase was also different. It can be concluded that the thermal release process was determined by the thickener and nucleating agent.

#### 3.2.3. Degree of Supercooling

**Figure 7:**
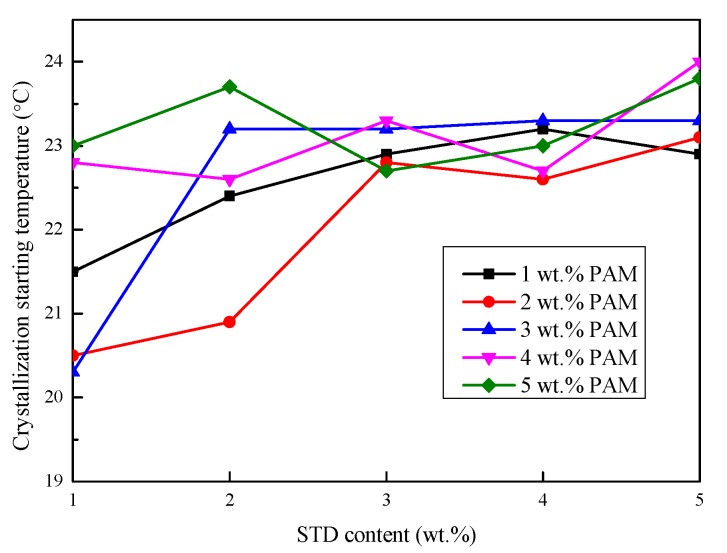
Effect of PAM and STD content on the crystallization starting temperature of the Na_2_SO_4_·10H_2_O composites.

**Figure 8:**
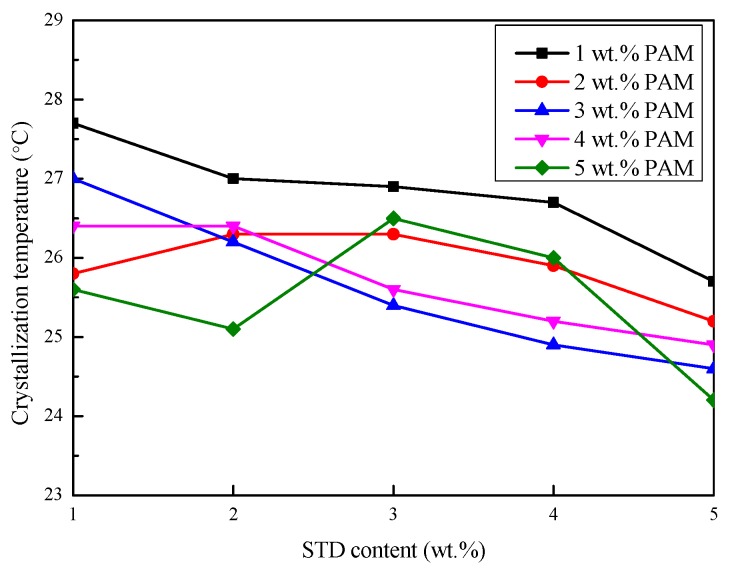
Effect of PAM and STD content on the crystallization temperature of the Na_2_SO_4_·10H_2_O composites.

**Figure 9:**
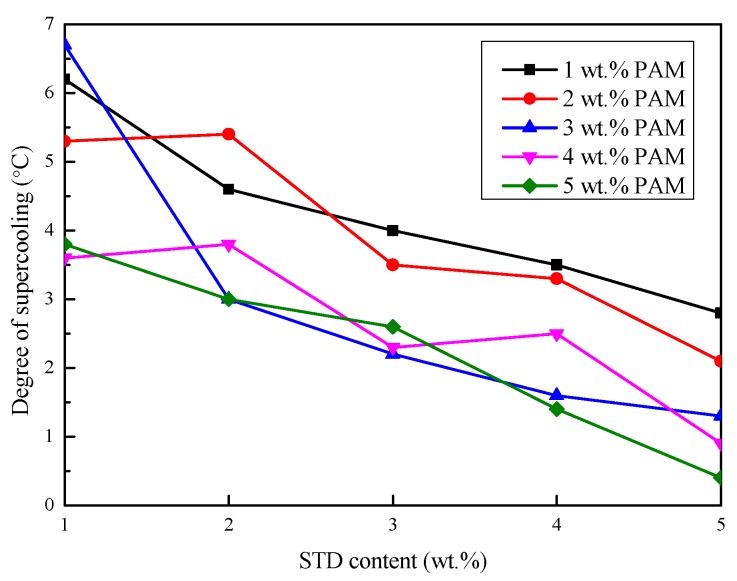
Effect of PAM and STD content on the degree of supercooling of the Na_2_SO_4_·10H_2_O composites.

The crystallization starting temperature, crystallization temperature, and the degree of supercooling of the Na_2_SO_4_·10H_2_O–5 wt.%KCl eutectics containing different proportions of PAM and STD were obtained from the cooling curves and are shown in Figure 7, Figure 8 and Figure 9. As shown in Figure 7, the crystallization starting temperature gradually increased with the increase in the STD content. Moreover, the smaller the PAM content, the greater was the extent of the increase. This was because more crystal nuclei were provided with the proportion of nucleating agent increased, which led to nucleation power being reduced and the crystallization being completed at a relatively high temperature. It can be seen from Figure 8 that the crystallization temperature of the eutectics reduced with the STD mass fraction increase, to varying degrees. This was because a new eutectic system was formed by the addition of STD, resulting in a decrease in the solidification temperature [29]. It can be seen from Figure 9 that the degree of supercooling of the Na_2_SO_4_·10H_2_O composites gradually decreased as the PAM and STD content increased. This was because the crystallization starting temperature increased and the crystallization temperature decreased as the PAM and STD content increased, and the degree of supercooling was effectively reduced. When the proportion of PAM and STD reached 5 wt.%, the subcooling of the composites reduced to 0.4 °C, which indicated that STD as nucleating agent could effectively suppress the supercooling of Na_2_SO_4_·10H_2_O.

### 3.3. Thermal Performance of Na_2_SO_4_·10H_2_O Composites with EG

#### 3.3.1. Thermal Conductivity

**Figure 10:**
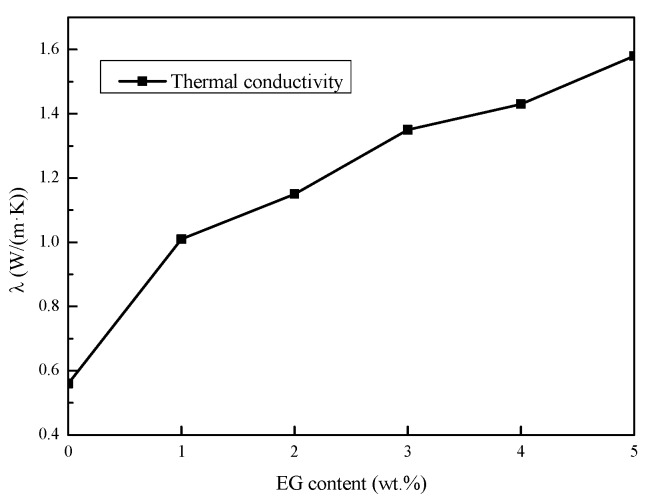
Thermal conductivities of the Na_2_SO_4_·10H_2_O composites with varying contents of expanded graphite (EG).

Through the analysis above, the sample S-5-5 showed a perfect thermal performance without the phase separation phenomenon, and the degree of supercooling was only 0.4 °C. However, the thermal conductivity of the composites was not enough. The heat transfer performance is further improved by the EG addition. Figure 10 shows the thermal conductivity of the Na_2_SO_4_·10H_2_O composites with varying contents of EG. It can be observed from the figure that the thermal conductivity of the composites increased continuously as the proportion of EG increased. Compared to the sample S-5-5 without EG (0.56 W/(m·K)), the thermal conductivity of the Na_2_SO_4_·10H_2_O composites with 5 wt.% EG reached 1.58 W/(m·K), an increase of nearly 2.8 times. This was because the composites could be absorbed by EG, and the heat was conducted directly through the network formed by EG instead of the Na_2_SO_4_·10H_2_O.

#### 3.3.2. Cooling Curves

**Figure 11:**
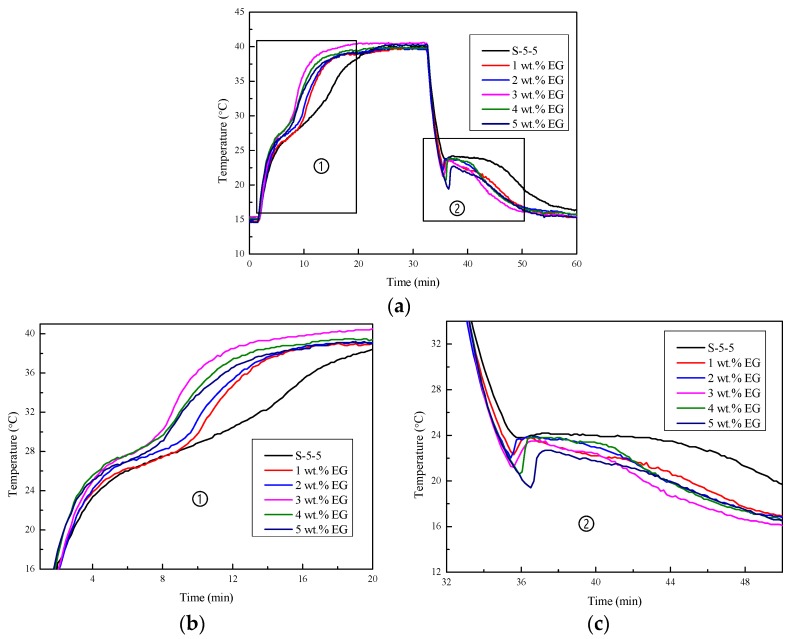
Cooling curves of Na_2_SO_4_·10H_2_O composites with varying contents of EG: (**a**) whole cooling curves; (**b**) enlargement of the heating process; (**c**) enlargement of the cooling process.

Figure 11 shows the cooling curves of Na_2_SO_4_·10H_2_O composites with varying contents of EG. From Figure 11 and the partial enlargement of the heating process (Figure 11b), it can be seen that the slope of the temperature curve became steeper as the EG content increased. This was because the conductivities of the CPCMs with a larger content of EG were higher. From the partial enlargement of the cooling process (Figure 11c), it can be seen that the addition of EG improved the heat release rate of the CPCMs at the same time. However, it also brought about some problems, such as subcooling. The supercooling degrees of the CPCMs with 1–5 wt.% EG addition were 1.4, 1.9, 2.3, 3.2, and 3.3 °C, respectively. This is because EG is a kind of porous medium, which can form a package for the Na_2_SO_4_·10H_2_O composites and limit the free movement of the inorganic hydrated salt molecules, thus affecting the aggregation and crystallization.

#### 3.3.3. DSC Curves

**Figure 12:**
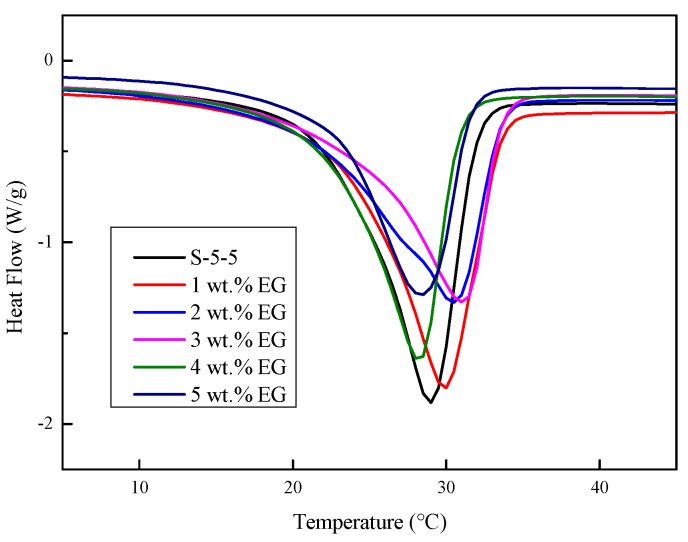
DSC curves of Na_2_SO_4_·10H_2_O composites with varying contents of EG.

The DSC curves of the Na_2_SO_4_·10H_2_O composites with varying contents of EG are shown in Figure 12, and the corresponding thermal data is shown in Table 3. It can be concluded that the melting temperatures of the CPCMs decreased as the EG increased. On the one hand, the thermal conductivities and the heat transfer efficiency of the CPCMs improved as the EG content increased. On the other hand, the integrity of the crystal structure was destroyed by the EG, which caused Na_2_SO_4_·10H_2_O crystal to be destroyed and melting to occur at lower temperature [30]. At the same time, the enthalpy of CPCMs was gradually reduced with the increase in EG. This was due to the decrease in the proportion of Na_2_SO_4_·10H_2_O. Additionally, the addition of EG led to a decrease in the crystallinity value (*CV*). The crystallinity value was proposed to reflect the effect of additives on the enthalpy of PCM and was calculated by Equation (1) [31]:

(1) $$CV=\frac{\Delta H_{n}}{\Delta H_{0}\left(1-n\%\right)}\;\times 100\%\;$$

where *CV* is the crystallinity value; *n* is the proportion of EG in the CPCMs; Δ*H*_0_ is the enthalpy of the CPCMs without EG; and Δ*H_n_* is the enthalpy of the CPCMs containing *n* wt.% EG.

In order to investigate the effect of EG on the enthalpy of the CPCMs, the CV of the sample S-5-5 without EG was set to 100%. It can be seen from Table 3 that the CV continuously decreased with the increase in EG content. This was because the steric effect and drag effect led to an increase in the lattice defect and reduced the possibility of crystallization, which caused the CV to not reach the ideal value [32]. When the content of EG exceeded 3 wt.%, the CV of the CPCMs was less than 85% because a part of the composite phase change material was adsorbed in the porous structure of EG, and the rest adhered to the surface of EG or was wrapped by EG. This was because the Na_2_SO_4_·10H_2_O molecules could not move freely due to forces such as surface tension, resulting in difficulty in crystallization and a decrease in enthalpy [30].

### 3.4. Thermal Reliability of Na_2_SO_4_·10H_2_O Eutectic CPCMs

#### 3.4.1. Ambient Temperature

**Figure 13:**
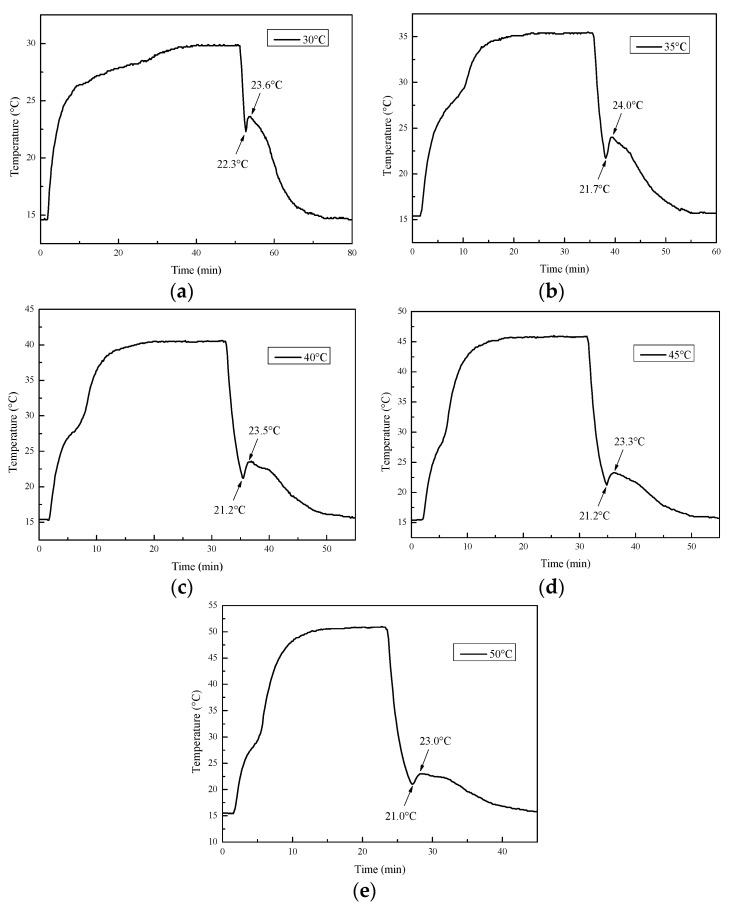
Cooling curves of the Na_2_SO_4_·10H_2_O eutectic composite phase change materials (CPCMs) at different ambient temperatures: (**a**–**e**) 30–50 °C.

**Figure 14:**
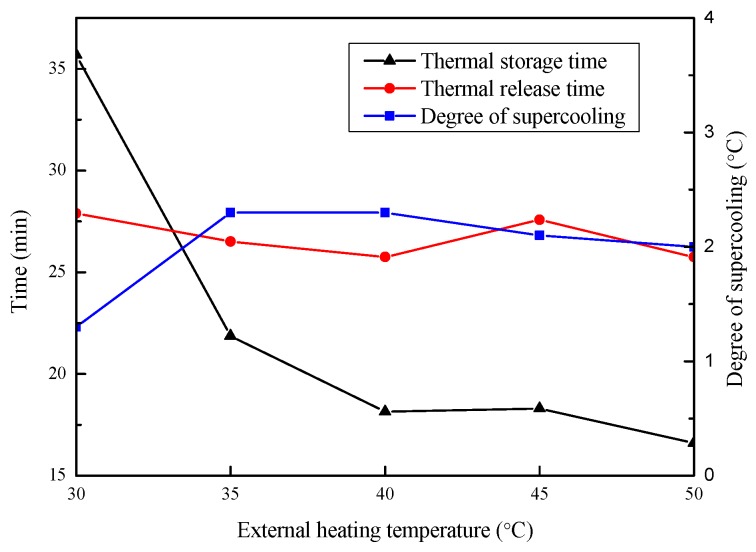
Effect of ambient temperature on the thermal storage and release time and the subcooling of the Na_2_SO_4_·10H_2_O eutectic CPCMs.

In order to explore the influence of ambient temperature on Na_2_SO_4_·10H_2_O eutectics, the heating temperature of the metal bath was set to 30, 35, 40, 45, and 50 °C. The cooling curves of the Na_2_SO_4_·10H_2_O eutectic CPCMs at different temperature are presented in Figure 13, and the corresponding thermal storage and release times, as well as the subcooling, are shown in Figure 14. It can be seen from Figure 14 that the heat storage time gradually decreased as the ambient temperature increased, indicating that increasing the ambient temperature had a significant impact on accelerating the melting rate and shortening the thermal storage time. The metal bath was kept at 15 °C during the cooling process, so the thermal release time remained substantially unchanged. The degree of supercooling of the CPCMs increased first and then remained stable with the increase in the ambient temperature and was maintained below 2.3 °C. This indicates that Na_2_SO_4_·10H_2_O eutectic CPCM has good thermal stability and be adapted to most outdoor environmental temperatures.

#### 3.4.2. Thermal Cycling Stability

**Figure 15:**
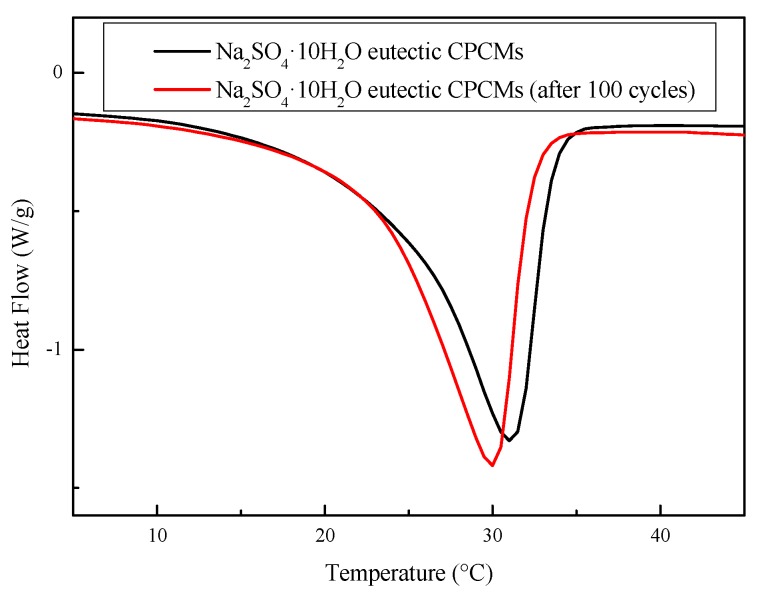
DSC curves of the Na_2_SO_4_·10H_2_O eutectic CPCMs before and after 100 melting–freezing cycles.

**Figure 16:**
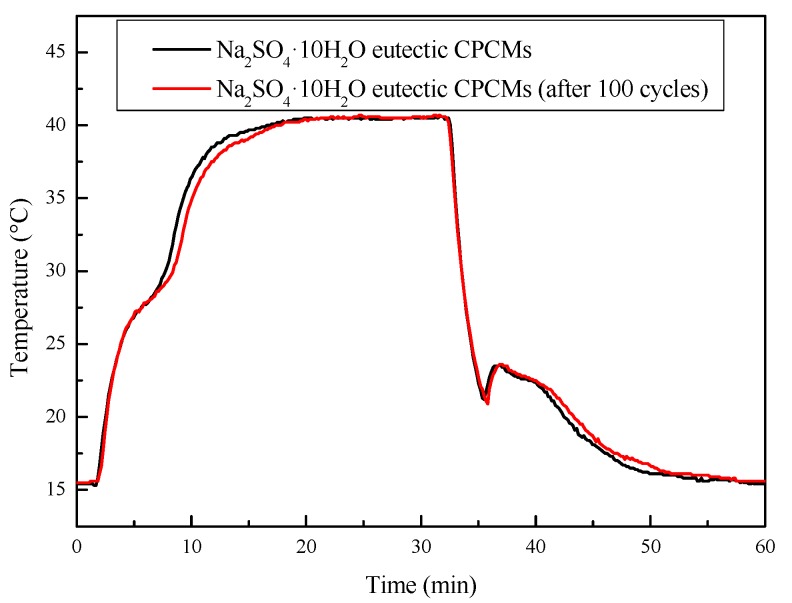
Cooling curves of the Na_2_SO_4_·10H_2_O eutectic CPCMs before and after 100 melting–freezing cycles.

Inorganic hydrated salt as PCMs have problems such as enthalpy decay and nucleating agent failure in long-term use. The thermal melting–freezing test was performed to determine the thermal cycling stability of the Na_2_SO_4_·10H_2_O eutectic CPCMs. The DSC curve and the cooling curve of the Na_2_SO_4_·10H_2_O eutectic CPCMs before and after 100 melting–freezing cycles are shown in Figure 15 and Figure 16, respectively. The melting temperature and enthalpy of Na_2_SO_4_·10H_2_O eutectic CPCMs before and after thermal cycling were 23.6 °C and 22.5 °C, and 111.3 J/g and 104.1 J/g, respectively. The degree of supercooling of the Na_2_SO_4_·10H_2_O eutectic CPCMs increased from 2.3 °C to 2.7 °C. This is because the nucleating agent STD itself is also an inorganic hydrated salt and may experience water loss during the reciprocating heating–cooling cycle test, resulting in a certain degree of nucleation effect but within a reasonable range. Through the analysis above, it can be concluded that the Na_2_SO_4_·10H_2_O eutectic CPCMs possess good thermal cycle stability.

#### 3.4.3. TGA Analysis

**Figure 17:**
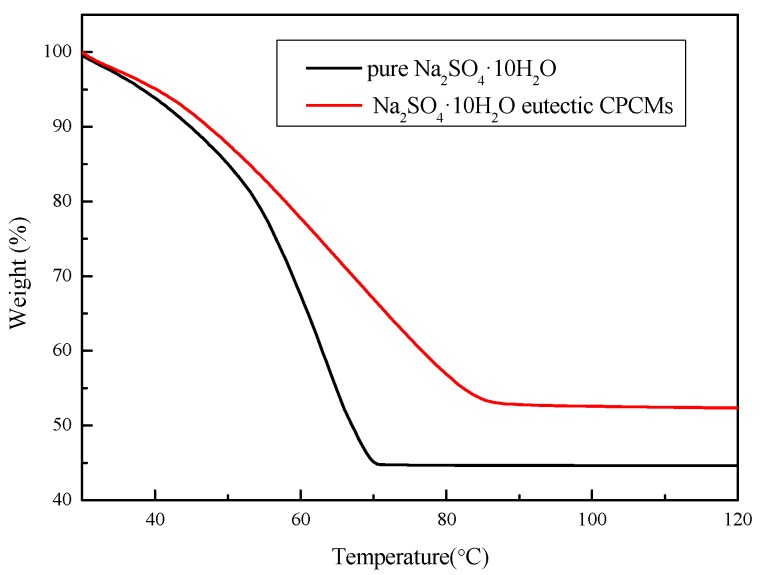
TGA curves of the pure Na_2_SO_4_·10H_2_O and Na_2_SO_4_·10H_2_O eutectic CPCMs.

Figure 17 shows the TGA curves of the pure Na_2_SO_4_·10H_2_O and Na_2_SO_4_·10H_2_O eutectic CPCMs. According to the TGA test results, the water loss rate of the pure Na_2_SO_4_·10H_2_O was 55.41%, and the theoretical calculation value was 55.90%; and the error was 0.8%. This was because Na_2_SO_4_·10H_2_O is highly prone to loss of water in the air, causing the actual test value to be slightly lower than the theoretical calculation value. The water loss rate of the Na_2_SO_4_·10H_2_O eutectic CPCMs was 47.63%, and the weight loss rate of the Na_2_SO_4_·10H_2_O eutectic CPCMs was lower than that of pure Na_2_SO_4_·10H_2_O. This was because additives such as EG have an adsorption and encapsulation effect on the PCM, making it difficult for the Na_2_SO_4_·10H_2_O eutectic CPCMs to lose water.

## 4. Conclusions

In this paper, a series of low-temperature eutectic CPCMs consisting of Na_2_SO_4_·10H_2_O and KCl were prepared. The Na_2_SO_4_·10H_2_O–5 wt.%KCl eutectics were modified by adding PAM, STD, and EG. Then, the phase separation condition, cooling curve, DSC curve, and thermal conductivity of the Na_2_SO_4_·10H_2_O eutectic CPCMs were explored. The thermal reliability of the Na_2_SO_4_·10H_2_O eutectic CPCMs were tested, including the ambient temperature test, thermal cycling stability test, and TGA analysis. The main conclusions are as follows:

1. The melting temperature of the Na_2_SO_4_·10H_2_O–KCl mixtures decreased as the KCl mass fraction increased. However, KCl addition could not improve the phase separation and supercooling situation of the eutectics.
2. In Na_2_SO_4_·10H_2_O–5 wt.% KCl eutectics with 5 wt.% PAM and 5 wt.% STD, almost no phase separation occurred, and the degree of supercooling reduced to 0.4 °C.
3. The results showed that the suitable mass fraction of EG was 3%, and the thermal conductivity increased to 1.35 W/(m·K), approximately 2.4 times that of the pure Na_2_SO_4_·10H_2_O.
4. The Na_2_SO_4_·10H_2_O–5 wt.% KCl eutectic CPCMs containing 5 wt.% PAM, 5 wt.% STD, and 3 wt.% EG showed perfect thermal reliability. After 100 thermal cycles, the enthalpy of the CPCMs remained at 104.1 J/g, and the degree of supercooling remained below 2.7 °C. The CPCMs could adapt to most outdoor environmental temperatures (as high as 50 °C), which indicates that the materials can be applied to building energy saving projects and improve personal thermal comfort.

## Figures and Tables

**Table 1:** Na_2_SO_4_·10H_2_O–5 wt.% KCl composites with varying contents of polyacrylamide (PAM) and sodium tetraborate decahydrate (STD).

Samples	PAM and STD Contents		Samples	PAM and STD Contents	
	PAM (wt.%)	STD (wt.%)		PAM (wt.%)	STD (wt.%)
S-0-0	0	0	S-3-3	3	3
S-1-1	1	1	S-3-4	3	4
S-1-2	1	2	S-3-5	3	5
S-1-3	1	3	S-4-1	4	1
S-1-4	1	4	S-4-2	4	2
S-1-5	1	5	S-4-3	4	3
S-2-1	2	1	S-4-4	4	4
S-2-2	2	2	S-4-5	4	5
S-2-3	2	3	S-5-1	5	1
S-2-4	2	4	S-5-2	5	2
S-2-5	2	5	S-5-3	5	3
S-3-1	3	1	S-5-4	5	4
S-3-2	3	2	S-5-5	5	5

**Table 2:** Thermal properties of the Na_2_SO_4_·10H_2_O–KCl eutectic mixtures with varying contents of KCl.

KCl Contents	Melting Temperature (°C)	Enthalpy (J/g)	Crystallization Starting Temperature (°C)	Crystallization Temperature (°C)	Degree of Supercooling (°C)
Pure	33.1	249.4	16.3	30.9	14.6
1 wt.%	29.9	145.5	13.4	30.3	16.9
3 wt.%	27.8	140.9	12.9	28.6	15.7
5 wt.%	25.3	128.7	10.5	26.2	15.7
7 wt.%	24.6	82.7	8.3	24.7	16.4

**Table 3:** Thermal properties of Na_2_SO_4_·10H_2_O composites with varying contents of EG.

EG Contents	Melting Temperature (°C)	Enthalpy (J/g)	CV (%)
S-5-5	23.9	128.2	100
1 wt.%	23.8	127.8	99.7
2 wt.%	22.3	117.4	91.6
3 wt.%	23.6	111.3	86.8
4 wt.%	22.5	106.8	83.3
5 wt.%	22.8	95.2	74.3
